# Comparison of mortality prediction models in acute respiratory distress syndrome undergoing extracorporeal membrane oxygenation and development of a novel prediction score: the PREdiction of Survival on ECMO Therapy-Score (PRESET-Score)

**DOI:** 10.1186/s13054-017-1888-6

**Published:** 2017-12-12

**Authors:** Michael Hilder, Frank Herbstreit, Michael Adamzik, Martin Beiderlinden, Markus Bürschen, Jürgen Peters, Ulrich H. Frey

**Affiliations:** 1 Klinik für Anästhesiologie und Intensivmedizin, Universität Duisburg-Essen, and Universitätsklinikum Essen, Hufelandstraße 55, 45147 Essen, Germany; 2 Klinik für Anästhesiologie und Intensivmedizin, Marienhospital Osnabrück, Osnabrück, Germany

**Keywords:** Acute respiratory distress syndrome, Extracorporeal membrane oxygenation, Mortality, Prediction, Survival

## Abstract

**Background:**

Extracorporeal membrane oxygenation (ECMO) is a life-saving therapy in acute respiratory distress syndrome (ARDS) patients but is associated with complications and costs. Here, we validate various scores supposed to predict mortality and develop an optimized categorical model.

**Methods:**

In a derivation cohort, 108 ARDS patients (2010–2015) on veno-venous ECMO were retrospectively analysed to assess four established risk scores (ECMOnet-Score, RESP-Score, PRESERVE-Score, Roch-Score) for mortality prediction (receiver operating characteristic analysis) and to identify by multivariable logistic regression analysis independent variables for mortality to yield the new PRESET-Score (PREdiction of Survival on ECMO Therapy-Score). This new score was then validated both in independent internal (*n* = 82) and external (*n* = 59) cohorts.

**Results:**

The median (25%; 75% quartile) Sequential Organ Failure Assessment score was 14 (12; 16), Simplified Acute Physiology Score II was 62.5 (57; 72.8), median intensive care unit stay was 17 days (range 1–124), and mortality was 62%. Only the ECMOnet-Score (area under curve (AUC) 0.69) and the RESP-Score (AUC 0.64) discriminated survivors and non-survivors. Admission pH_a_, mean arterial pressure, lactate, platelet concentrations, and pre-ECMO hospital stay were independent predictors of death and were used to build the PRESET-Score. The score’s internal (AUC 0.845; 95% CI 0.76–0.93; *p* < 0.001) and external (AUC 0.70; 95% CI 0.56–0.84; *p* = 0.008) validation revealed excellent discrimination.

**Conclusions:**

While our data confirm that both the ECMOnet-Score and the RESP-Score predict mortality in ECMO-treated ARDS patients, we propose a novel model also incorporating extrapulmonary variables, the PRESET-Score. This score predicts mortality much better than previous scores and therefore is a more precise choice for decision support in ARDS patients to be placed on ECMO.

## Background

Acute respiratory distress syndrome (ARDS) remains a life-threatening disease with mortality up to 55% [1], and supportive care by prone positioning [2] and protective lung ventilation [3] improve survival. However, even with nitric oxide inhalation, or intravenous steroids, many patients still suffer from critical arterial hypoxaemia and hypercarbia [4].

Extracorporeal membrane oxygenation (ECMO) therapy has received substantial and renewed interest due to improvements in ECMO technology and safety, with increased survival suggested by the CESAR trial, favourable outcome in A(H1N1) influenza-evoked ARDS, and indication of avoidance of substantial lung and organ injury with early ECMO [5–8]. As a result, ECMO is now implemented more frequently and in a broader spectrum of patients.

However, ECMO demands specialized personnel and techniques, and can be associated with severe complications. Therefore, appropriate patient selection based on outcome prediction by scoring may help in decision-making. Several scoring systems have been proposed such as the ECMOnet-Score, developed for risk stratification in H1N1 pneumonia [6], the RESP-Score including data from more than 2000 patients [9], the Predicting Death for Severe ARDS on VV-ECMO (PRESERVE)-Score [10], the Roch-Score [11], and the Enger-Score [12].

However, limitations of these previous scores include usage of different ECMO technologies and procedures, patient heterogeneity, and also statistical methods which did not always take into account optimum validation of results [13, 14]. Moreover, for practical usefulness, any scoring system requires both categorization of risk variables and a limitation in the number of variables. In any case, external validation of published scores is mandatory before any scoring system can generally be accepted.

Accordingly, the aim of this study was to conduct an independent validation of the categorical ECMOnet-Score, the RESP-Score, the Roch-Score, and the PRESERVE-Score to investigate their usefulness in predicting survival in a single ECMO centre. Furthermore, we developed a novel and easy-to-use categorical score based on pre-ECMO clinical data, the PREdiction of Survival on ECMO Therapy-Score (PRESET-Score), to be used to facilitate decision-making and validated this score in two independent validation cohorts.

## Methods

### Study design

This non-interventional study was performed in agreement with the ethical principles and standards of the second Helsinki declaration and its later amendments. This retrospective study did not need ethical approval, which was waived by the ethics committee of the University Hospital Essen (AZ 15-6729-BO), as our data analysis precluded possible interference between this prospective observational study and the decisions regarding the patients’ clinical management.

#### Derivation cohort

Data were collected from 108 consecutive ARDS patients receiving vv-ECMO therapy in our intensive care unit (ICU) at the University Hospital Essen, Germany, between 30 December 2009 and 10 February 2015.

#### Internal validation cohort

Data were prospectively collected from a further 82 consecutive ARDS patients receiving vv-ECMO therapy in the ICU of the University Hospital Essen, Germany, between 11 February 2015 and 9 January 2017.

#### External validation cohort

Data were collected from 59 ARDS patients receiving vv-ECMO therapy in the ICU of the Marienhospital Osnabrück, Germany, between 23 February 2013 and 26 December 2015.

#### ECMO therapy regime

After referral of patients for ECMO therapy, a conservative treatment protocol consisting of prone positioning, optimized ventilator settings, and exclusion of reversible conditions (pneumothorax) was performed. Each patient in the derivation and the validation groups was ventilated with biphasic positive airway pressure (BIPAP) prior to ECMO initiation. ECMO was initiated at the referring hospital by our retrieval team if transport without extracorporeal support was not feasible. No single cut-off value was used to make this decision. ECMO was initiated when hypoxaemia and/or profound hypercarbia with haemodynamic instability persisted despite optimized treatment, as reported previously [8]. Contraindications for vv-ECMO were cardiogenic shock and terminal pulmonary disease with no prospect for lung transplantation in the near future. Eighty-six per cent of patients were cannulated at the referring hospital and transported with ECMO.

vv-ECMO was initiated by performing percutaneous cannulations, preferably bi-femoral venous cannulation, with a minimum distance of 15 cm between cannulae tips to prevent recirculation of oxygenated blood. The circuit configuration was as follows: HLS cannulae (19–25 F depending on patient size) (Maquet, Getinge Group, Rastatt, Germany), heparin-coated (Bioline) tubing (Cardiohelp HLS Set 7.0; Maquet), centrifugal pump, oxygenator, and heater (HU35; Maquet).

At the beginning of ECMO treatment the ECMO pump speed was set to achieve maximum flow. A Swan–Ganz catheter was placed in all patients on admission to the ICU. After determination of cardiac output, the target ECMO flow was set in order to keep the SaO_2_ over 90% and the ratio of ECMO flow to cardiac output above 60%, which is supposed to be sufficient for adequate blood oxygenation [15]. Initially, 100% oxygen was used as the sweep gas. The sweep gas flow was adjusted to maintain normocapnia with protective ventilation.

### Data collection

Demographic and clinical characteristics as well as haemodynamic, respiratory, and physiologic variables were obtained immediately before initiation of ECMO therapy and data for hospital survival were recorded. The ECMOnet-Score [6], RESP-Score [9], PRESERVE-Score [10], and Roch-Score [11] were calculated according to the original publications.

### Statistical analysis

Data were tested for normal distribution using the Kolmogorov–Smirnov and Shapiro–Wilk tests. Continuous variables were presented as mean ± standard deviation (SD) in the case of normally distributed data or as median (MD), first quartile (Q1), and third quartile (Q3) in the case of not normally distributed data. Analysis was carried out using Student’s *t* test or the Mann–Whitney *U* test, as appropriate. Categorical variables were recorded, frequency percentages were calculated, and the χ^2^ test was used for these analyses. Each score’s discriminatory power was assessed by calculating the area under the receiver operating characteristic (ROC) curve (AUC), and calibration was evaluated using the Hosmer–Lemeshow goodness-of-fit *C* test [16]. In this test, *p* > 0.05 suggests good calibration. Comparison of ROC curves was carried out using the method described by Hanley and McNeil [17].

Variables relating to patients, diagnoses, or associated organ dysfunction prior to ECMO initiation were considered. Univariate comparison of all variables in survivors and non-survivors was undertaken. Variables with *p* ≤ 0.1 in univariate analysis were entered into a backward stepwise binary logistic regression model to identify candidate variables for inclusion in the PRESET-Score after exploration of linearity. Non-significant variables were removed at each step. Linear variables were converted into categorical variables based on Wald statistics for weighting variables, with groups of similar quantity using the relative contribution of each beta parameter [1].

In the “internal validation set”, binary logistic regression was used to reassess the PRESET-Score performance in an independent data internal dataset. Model discrimination and calibration were assessed using the area under the ROC curve and the Hosmer–Lemeshow *C* statistic with associated *p* value, respectively.

The “external validation set” consisted of a completely independent cohort after building the PRESET-Score from the derivation cohort. The external validation of the PRESET-Score was performed on a dataset of 59 patients from the Marienhospital Osnabrück, Osnabrück, Germany (external validation cohort).

Statistical analyses were performed using SPSS (V 22.0; SPSS Inc., Chicago, IL, USA).

## Results

### Patient characteristics

The derivation cohort’s demographic and clinical pre-ECMO characteristics are presented in Table 1. The median (25%; 75% quartile) Sequential Organ Failure Assessment (SOFA) score was 14 (12; 16) and the Simplified Acute Physiology Score II (SAPS II) was 62.5 (57; 72.8). The median ICU stay was 17 days (range 1–124) and mortality was 62%. The leading primary causes of ARDS were bacterial (45%) and H1N1 (19%) pneumonia, respectively.

**Table 1:** Demographic characteristics of patients before ECMO stratified for survival and non-survival

	Total (*n* = 108)		Survivors (*n* = 41)		Non-survivors (*n* = 67)		*p* value
Age (years)	48	(37; 55)	47	(36; 55)	48	(38; 56)	0.421
Sex, male (%)	69	(64)	27	(66)	42	(63)	0.739
Weight (kg)	85	(70; 100)	90	(80; 103)	82	(70; 100)	0.050
Height (m)	1.75	(1.69; 1.8)	1.75	(1.7; 1.8)	1.75	(1.66; 1.8)	0.393
Body mass index (kg m^–2^)	27.8	(24.5; 32.0)	29.3	(26.3; 33.8)	26.3	(24.2; 30.9)	0.028
Immunocompromised (%)^a^	41	(38)	11	(26.8)	30	(44.8)	0.062
Cirrhosis (%)	12	(11.1)	4	(9.8)	8	(11.9)	
Solid cancer (%)	10	(9.3)	2	(4.9)	8	(11.9)	
Haematological malignancies (%)	8	(7.4)	4	(9.8)	4	(6.0)	
Long-term corticosteroids (%)	5	(4.6)	0	(0.0)	5	(7.5)	
Solid organ transplantation (%)	3	(2.8)	1	(2.4)	2	(3.0)	
AIDS (%)	3	(2.8)	0	(0.0)	3	(4.5)	
CNS dysfunction (%)^b^	39	(36.1)	13	(31.7)	26	(38.8)	0.456
Bicarbonate infusion (%)	70	(64.8)	24	(58.5)	46	(68.7)	0.285
Cardiac arrest (%)	22	(20.4)	7	(17.1)	15	(22.4)	0.506
SOFA score	14	(12; 16)	13	(11; 15)	15	(12; 17)	< 0.001
SAPS II	63	(57; 73)	60	(56; 68)	65	(59; 75)	0.009
Prone positioning (%)	53	(49.1)	19	(46.3)	34	(50.7)	0.657
Neuromuscular blocker use (%)	103	(95.4)	40	(97.6)	63	(94)	0.397
Acute respiratory failure diagnostic groups							
Bacterial pneumonia (%)	45	(41.7)	20	(48.8)	25	(37.3)	0.241
H1N1 pneumonia (%)	19	(17.6)	7	(17.1)	12	(17.9)	0.912
Pre-ECMO ventilator settings							
PEEP (mbar)	15	(12; 18)	15	(12; 18)	15	(14; 18)	0.420
PIP = P_plat_ (mbar)	34.5	(30; 38)	34	(30.5; 35)	35	(30; 38)	0.384
PaO_2_/FiO_2_ (mmHg)	72	(58; 91)	71	(60; 94)	73	(55; 91)	0.529
F_i_O_2_ (%)	100	(92.5; 100)	100	(80; 100)	100	(100; 100)	0.058
Mean airway pressure (mbar)	25	(22; 27)	25	(22; 26.5)	25	(22; 28)	0.231
Minute ventilation (l min^–1^)	9.2	(2.6)	9.3	(2.6)	9.2	(2.6)	0.829
Respiratory rate (min^–1^)	20	(19; 24)	20	(19.5; 23.5)	20	(19; 24)	0.860
Interval MV-ECMO (h)	37	(17.3; 102)	26	(17; 84.5)	40	(19; 124)	0.140
Pre-ECMO blood gases							
PaCO_2_ (mmHg)	65	(54; 84)	66	(46; 75)	65	(59; 88)	0.201
pH_a_	7.20	(0.12)	7.24	(0.13)	7.17	(0.11)	0.005
PaO_2_ (mmHg)	68	(57; 81)	66	(58; 80)	69	(55; 84)	0.674
SaO_2_ (%)	91	(85.1; 94)	91	(86.9; 93)	90.6	(85; 94.3)	0.641
Haemoglobin concentration (g dl^–1^)	10.4	(9.2; 12.0)	10.7	(9.6; 12.6)	10.1	(9.2; 11.2)	0.115
Lactate concentration (mmol l^–1^)	2.8	(1.5; 5.9)	2.3	(1.3; 3.4)	4.1	(1.6; 8.5)	0.001
Bilirubin concentration (mg dl^–1^)	0.7	(0.4; 1.4)	0.6	(0.4; 0.9)	1.0	(0.5; 1.7)	0.035
Creatinine concentration (mg dl^–1^)	1.2	(0.9; 2.2)	1.2	(0.9; 1.8)	1.3	(0.9; 2.5)	0.168
Haematocrit (%)	33.9	(31.3; 37)	34	(32; 37.7)	32.8	(31; 36.4)	0.182
CRP concentration (mg dl^–1^)	22.9	(15.0)	22.4	(16.8)	23.1	(13.8)	0.811
PCT concentration (ng ml^–1^)	4.7	(1.0; 24.9)	5.4	(0.5; 15.3)	4.1	(1.8; 32.7)	0.155
Platelet concentration (×1000 μl^–1^)	162	(90; 245)	209	(116; 286)	139	(69; 202)	0.005
Hemodynamic variables							
Heart rate (min^–1^)	115	(100; 130)	109	(90; 120)	120	(103; 130)	0.015
Mean arterial pressure (mmHg)	80	(72; 92)	87	(80; 104)	77	(70; 87)	< 0.001
Norepinephrine dosage (μg kg^–1^ min^–1^)	0.62	(0.22; 1.91)	0.3	(0.1; 0.67)	0.95	(0.44; 3.06)	< 0.001
Pre-ECMO hospital stay (days)	4	(2; 9)	3	(1; 6)	4	(2; 12)	0.060
Lung injury score	3.0	(3.0; 3.3)	3.0	(2.8; 3.3)	3.3	(3.0; 3.5)	0.106

Variables presented as mean (standard deviation) in the case of normally distributed data or as median (first quartile; third quartile) in the case of not normally distributed data

*ECMO* extracorporeal membrane oxygenation, *CRP* C-reactive protein, *PCT* procalcitonin, *MV* mechanical ventilation, *SOFA* Sequential Organ Failure Assessment, *SAPS* Simplified Acute Physiology Score, *PEEP* positive end-expiratory pressure, *PIP* peak inspiratory pressure, *P*_*plat*_ plateau pressure, *AIDS* acquired immune deficiency syndrome, *SaO*_*2*_ arterial oxygen saturation, *CNS*, central nervous system, *PaO*_*2*_ partial pressure of arterial oxygen, *FiO*_*2*_ fraction of inspired oxygen, *PaCO*_*2*_ partial pressure of carbon dioxide

^a^“Immunocompromised” defined as presence of haematologic malignancies, solid tumour, solid organ transplantation, human immunodeficiency virus, long-term corticosteroid treatment, or liver cirrhosis

^b^“CNS dysfunction” diagnosis combined neurotrauma, stroke, encephalopathy, cerebral embolism, seizure, and epileptic syndrome

Stratification of patient data according to the different prognostic scores is presented in Table 2. ROC analysis showed significant discrimination for the ECMOnet-Score (AUC 0.69; *p* = 0.001) and the RESP-Score (AUC 0.64; *p* = 0.012). In a subsample analysis including only H1N1 patients (*n* = 19), the ECMOnet-Score also performed very well (AUC 0.79; *p* = 0.045). Calibration exhibited similar performances based on the Hosmer–Lemeshow goodness-of-fit test for the ECMOnet-Score (χ^2^ = 3.7; *p* = 0.876), the RESP-Score (χ^2^ = 8.4; *p* = 0.395), and the PRESERVE-Score (χ^2^ = 5.2; *p* = 0.396), while the Roch-Score showed a weaker performance (Table 2).

**Table 2:** Assessment of prediction scores

	Total	Survivors	Non-survivors	AUC	95% CI	*p* value	Hosmer–Lemeshow statistic (χ^2^; *p* value)	Pairwise comparison to PRESET-Score (difference between areas; *p* value)
Derivation cohort (*n*)	108	41	67					
ECMOnet-Score	4.5 (3; 6)	4 (3; 5)	5 (4; 7)	0.695	0.59–0.79	0.001	3.7; 0.876	0.127; 0.0260
RESP-Score	–1 (–6; 3)	1 (–3; 4)	–2 (–7; 2)	0.645	0.53–0.75	0.012	8.4; 0.395	0.178; 0.0045
PRESERVE-Score	4 (3; 6)	4 (2; 5)	5 (3; 6)	0.593	0.48–0.71	0.106	5.2; 0.396	0.230; 0.0010
Roch-Score	3 (3; 4)	3 (2; 4)	3 (3; 4)	0.564	0.45–0.68	0.269	5.4; 0.020	0.259; 0.0003
PRESET-Score	7 (5; 10)	5 (4; 7)	8 (7; 11)	0.823	0.74–0.90	< 0.001	2.9; 0.940	
Internal validation cohort (*n*)	82	37	45					
PRESET-Score	6 (5; 10)	4 (4; 6)	8 (6; 11)	0.845	0.76–0.93	< 0.001	10.9; 0.207	
External validation cohort (*n*)	59	31	28					
PRESET-Score	7 (5; 9)	6 (4; 8)	8 (7; 10)	0.700	0.56–0.83	0.008	4.2; 0.655	

Variables presented as median (first quartile; third quartile)

*ARDS* acute respiratory distress syndrome, *AUC* area under the curve, *CI* confidence interval, *ECMO* extracorporeal membrane oxygenation, *PRESERVE-Score* Predicting Death for Severe ARDS on VV-ECMO, *PRESET-Score* PREdiction of Survival on ECMO Therapy-Score, *VV* veno-venous

### PREdiction of Survival on ECMO Therapy-Score (PRESET Score)

**Fig 1:**
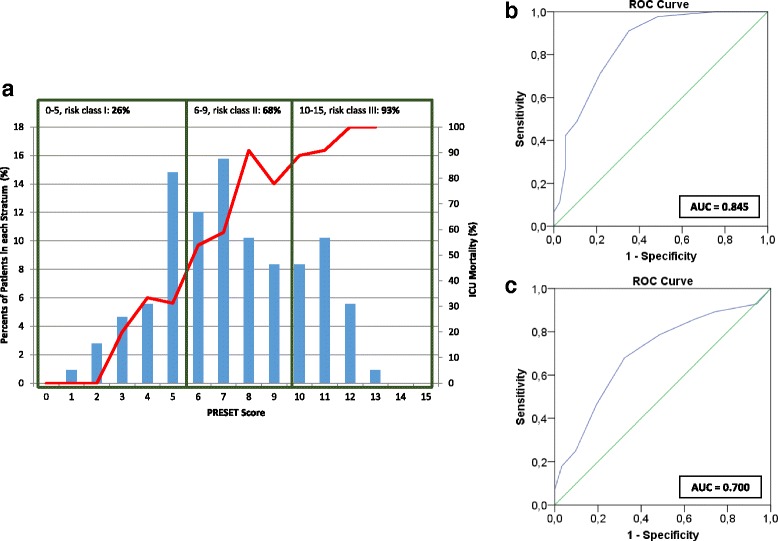
PRESET-Score in ARDS patients requiring ECMO therapy. **a** Distribution of values in relation to the observed ICU mortality rate (solid line) at each value. **b** ROC curve in the internal validation group (*n* = 82). **c** ROC curve in the external validation test set (*n* = 59). AUC area under the curve, ICU intensive care unit, PRESET-Score PREdiction of Survival on ECMO Therapy-Score, ROC receiver operating characteristic

We next performed a multivariate analysis using 11 variables from Table 1 with *p* ≤ 0.1 to identify factors independently associated with mortality, and identified five clinical variables independently associated with ICU death: high lactate concentration, more hospital days pre ECMO, low mean arterial pressure, low platelet count, and low arterial pH (Table 3). Categorization of these variables with weighting related to the Wald statistics and comparable contribution of similar quantities into groups resulted in the new PRESET-Score (Tables 3 and 4). Three risk classes were identified, namely class I (PRESET-Score 0–5, *n* = 31), class II (PRESET-Score 6–9, *n* = 50), and class III (PRESET-Score 10–15, *n* = 27) with corresponding ICU mortality of 26%, 68%, and 93%, respectively (Fig. 1a). Median (25%; 75% quartile) hospital mortality for class I was 58 days (31; undefined), for class II was 23 days (13; 40), and for class III was 12 days (1; 16).

**Table 3:** Results of multivariate analysis

Variable	Wald statistic	*p* value	HR	95% CI
pH_a_ (×10)	3.92	0.048	0.64	0.42–0.99
Hospital stay pre ECMO (days)	7.48	0.006	1.18	1.05–1.34
Lactate concentration (mmol l^–1^)	7.66	0.006	1.38	1.10–1.74
Mean arterial pressure (mmHg 10^–1^)	9.10	0.003	0.53	0.35–0.80
Platelet concentration (100,000 μl^–1^)	7.33	0.007	0.56	0.37–0.85

*HR* hazard ratio, *CI* confidence interval, *ECMO* extracorporeal membrane oxygenation

**Table 4:** PRESET-Score at ECMO initiation

Variable	Points
Mean arterial pressure (mmHg)	
> 100	0
91–100	1
81–90	2
71–80	3
≤ 70	4
Lactate concentration (mmol l^–1^)	
≤ 1.50	0
1.51–3.00	1
3.01–6.00	2
6.01–10.00	3
> 10.00	4
pH_a_	
> 7.300	0
7.201–7.300	1
7.101–7.200	2
≤ 7.100	3
Platelet concentration (×1000 μl^–1^)	
> 200	0
101–200	1
≤ 100	2
Hospital days pre ECMO	
≤ 2	0
3–7	1
> 7	2
Total score	0–15
ICU mortality by risk class	Mortality (%)
PRESET-Score 0–5, risk class I	26
PRESET-Score 6–9, risk class II	68
PRESET-Score 10–15, risk class III	93

*ECMO* extracorporeal membrane oxygenation, *ICU* intensive care unit, *PRESET-Score* PREdiction of Survival on ECMO Therapy-Score

ROC analysis displayed a significant discrimination with an AUC of 0.823 (95% CI 0.74–0.90; *p* < 0.001). Moreover, pairwise comparison with established scores demonstrated a better performance with differences ranging from 0.127 (ECMOnet-Score) to 0.259 (Roch-Score; Table 2).

We next used two independent validation cohorts to verify the findings from the derivation cohort. The internal validation cohort consisted of 82 patients; 51 (62%) patients were male and 31 (38%) patients were female. During the stay in our ICU, 45 (55%) patients died. The mean age of patients upon admission to our ICU was 50.3 years ± 13.9. The average duration of mechanical ventilation before ECMO was 59.9 hours ± 80.5. The average oxygenation index according to Horovitz was 82.6 mmHg ± 42.4 before ECMO connection and the average p_a_CO_2_ value was 66.5 mmHg ± 25.4, with a lowest value of 29 mmHg and a highest of 175 mmHg, respectively. The average SOFA score and SAPS II at the time of ECMO initiation were 14.6 ± 2.74 and 65.3 ± 12.3, respectively. Characteristics were not significantly different between the derivation and the internal validation cohorts. Prospective validation (*n* = 82; mortality 55%) demonstrated excellent discrimination (AUC 0.845; 95% CI 0.76–0.93; Table 2, Fig. 1b). Applying the above risk categories to the internal validation cohort yielded a mortality of 14% for class I (*n* = 28), 67% for class II (*n* = 33), and 91% for class III (*n* = 21) patients, respectively.

External validation comprising 59 patients showed a mortality of 47%. Demographic statistics in comparison with the internal validation cohort showed smaller SOFA score and SAPS II values as well as a higher oxygenation index and further differences regarding pH_a_, p_a_CO_2_, creatinine, pre-ECMO hospital days, as well as norepinephrine dosages at admission compared to the internal validation cohort (Table 5). Nevertheless, ROC analysis using the PRESET score also showed a good score performance (AUC 0.70; 95% CI 0.56–0.83; Table 2, Fig. 1c) and good calibration, with a Hosmer–Lemeshow chi-square of 4.2 (*p* = 0.655). Applying the above risk categories to the external validation cohort yielded a mortality of 27% for class I (*n* = 15), 50% for class II (*n* = 34), and 70% for class III (*n* = 10) patients, respectively.

**Table 5:** Characteristics of patients for the external and internal validation group before ECMO

	External validation group (*n* = 59)		Internal validation group (*n* = 82)		*p* value
Age (years)	56	(44; 63)	54	(40; 60)	0.247
Sex, male (%)	41	(69)	51	(62)	0.369
Weight (kg)	90	(80; 103)	92	(80; 120)	0.145
SOFA score	13	(11; 14)	14	(12; 17)	0.001
SAPS II	46	(41; 56)	64	(56; 73)	< 0.001
Pre-ECMO ventilator settings					
PEEP (mbar)	15	(14; 18)	15	(14; 17)	0.269
PIP = P_plat_ (mbar)	32	(30; 35)	34	(30; 36)	0.270
PaO_2_/FiO_2_ (mmHg)	106	(73; 175)	70	(57; 92)	< 0.001
Minute ventilation (l min^–1^)	9.2	(2.6)	9.6	(3.2)	0.736
Pre-ECMO blood gases					
PaCO_2_ (mmHg)	71	(21)	66	(25)	0.063
pH_a_	7.17	(0.14)	7.25	(0.13)	0.001
Lactate concentration (mmol l^–1^)	2.3	(1.3; 4.4)	2.2	(1.3; 5.3)	0.562
Bilirubin concentration (mg dl^–1^)	0.8	(0.4; 1.2)	0.9	(0.5; 1.6)	0.208
Creatinine concentration (mg dl^–1^)	1.1	(0.8; 1.8)	1.3	(0.9; 2.4)	0.026
Platelet concentration (×1000 μl^–1^)	146	(87; 195)	171	(102; 263)	0.145
Haemodynamic variables					
Mean arterial pressure (mmHg)	80	(16)	80	(14)	0.662
Norepinephrine dosage (μg kg^–1^ min^–1^)	0.6	(0.3; 0.9)	0.3	(0.1; 0.7)	0.002
Pre-ECMO hospital stay (days)	3	(1; 7)	4	(3; 8)	0.040
Hospital mortality, *n* (%)	28	(47)	45	(55)	

Variables presented as mean (standard deviation) in the case of normally distributed data or as median (first quartile; third quartile) in the case of not normally distributed data

*ECMO* extracorporeal membrane oxygenation, *MV* mechanical ventilation, *SOFA* Sequential Organ Failure Assessment, *SAPS* Simplified Acute Physiology Score, *PEEP* positive end-expiratory pressure, *PIP* peak inspiratory pressure, *P*_*plat*_ plateau pressure, *PaO*_*2*_ partial pressure of arterial oxygen, *FiO*_*2*_ fraction of inspired oxygen, *PaCO*_*2*_ partial pressure of carbon dioxide

## Discussion

Acute respiratory distress syndrome (ARDS) is frequently fatal and ECMO is currently considered over a wide range of indications from a last therapeutic resort to a protective and perhaps even “prophylactic” therapy. Accordingly, there is much discussion and controversy about the indications/contraindications of ECMO and the time of initiation. In any case, prognostic systems should enable outcome prediction of such a therapy and the ECMOnet-Score, RESP-Score, PRESERVE-Score, and ROCH-Score have all served to improve such a prediction.

However, for any prognostic system to be generally applicable, it is essential to validate this system in at least one independent cohort.

We therefore not only evaluated these latter previously proposed predictive scores, but also generated and validated a new score, based on pre-ECMO clinical variables, the PRESET-Score, and prospectively validated this score in two independent cohorts.

Interestingly, in the context of this new score derived from clinical variables immediately before ECMO initiation, only extrapulmonary variables were identified as predictors, namely mean arterial blood pressure, platelet concentration, pH_a_, lactate concentration, and hospital stay before ECMO therapy. Of note, respiratory variables themselves were not predictive of survival.

### Platelet concentration

As does the Sepsis-related Organ Failure Assessment (SOFA) score [18], our data support the independent prognostic value of the platelet count. In our patients, a decrease in platelets by 100,000 μl^–1^ increases mortality by 30%.

This result is supported by similar observations in critically ill patients and ECMO patients [19–21].

In this context, it should be noted that there may be an effect on the platelet count by the high number of potentially immunocompromised patients. While interaction of these two factors cannot be ruled out, multivariate analysis including presumed immunocompromised patients as a covariate demonstrated a higher weighting of “platelet count” and, therefore, remained in the final model.

### Lactate concentration

Lactate concentration is an established prognostic marker in ICU patients, and a concentration ≥ 4 mmol l^–1^ at admission increased mortality by sixfold within the first 3 days [22]. Comparable results were seen in 830 patients with severe sepsis admitted to an emergency department of a tertiary-care academic centre [23].

Concerning ECMO therapy, lactate has been shown to independently predict mortality when measured before ECMO initiation [24].

These results are in line with our findings. In our cohort, an initial lactate concentration ≥ 4 mmol l^–1^ was associated with a 5.7 times greater mortality, with an increase by 1 mmol l^–1^ increasing mortality by 30%.

### pH_a_

A multi-centre database comprising 1473 adult ARDS patients with ECMO therapy demonstrated a significant influence of pH_a_ on outcome [25], with a median pH_a_ of 7.29 in survivors, but 7.26 in non-survivors. Moreover, pH < 7.18 was associated with a 2.5-fold increased mortality compared to a subgroup with pH_a_ > 7.36. In our study, a decrease in pH by 0.1 was found to be associated with an increase in mortality by 40%.

### Hospital stay pre ECMO

The timing of ECMO initiation was and is a matter of debate. In a joint study by a French hospital and two Australian hospitals [26] the time from ICU admission to ECMO initiation was an independent predictor of death, and this was confirmed by a Swiss study [27]. In our study, each additional hospital day before ECMO initiation was associated with a 10% increase in mortality. One possible explanation might be that any ventilator day before ECMO may increase lung trauma and promotes multiple organ failure.

### Mean arterial pressure

While intuitively an association between decreased arterial pressure and mortality may be assumed, various recent studies have not confirmed such a specific correlation [28, 29]. Nevertheless, targeting a decreased arterial pressure is associated with an increased incidence of acute kidney injury [30, 31].

In our cohort, average mortality of 62% was greater when compared to other prediction models—that is, 32–56% in the studies comprising the ECMOnet-Score [6], ENGER-Score [12], PRESERVE-Score [10], RESP-Score [9], and ROCH-Score [11].

However, an unreflecting direct comparison of the mortality rates in ECMO patients in the different studies may neglect a bias since neither the practical implementation of ECMO nor the indication for its initiation is subject to a specific internationally or nationally standardized protocol.

One possible explanation for the lower survival rate of 38% may be due to ECMO initiation in patients who may have been excluded from ECMO therapy or are associated with a poor outcome when ELSO criteria [32] or other criteria [33] would be taken into account. For example, 41 (38%) patients in whom ECMO therapy was initiated may be considered immunocompromised, as suggested by their past medical history (see Table 1).

Our institution traditionally employed liberal inclusion criteria even in patients with a high probability of non-survival. On the other hand, a large proportion of patients explicitly referred to our centre for ECMO therapy were salvaged without ECMO. Thus, we feel that patients treated on ECMO at our centre tend to be rather sick. While this aggressive approach provides a potential rescue therapy for very sick patients with a low chance of survival, it consequently results in ECMO therapy at our centre being associated with a less positive outcome.

These considerations are reflected by higher SOFA score and SAPS II in the patients of the present study, which correlated well with mortality in different studies and settings [34, 35]. In fact, cohorts from other prognostic models as well as the external validation cohort had a much lower SOFA score and SAPS II in comparison to our patients, potentially explaining different survival rates (Table 5). Differences in the patients’ conditions, therefore, might also be an explanation for a poor performance of the other prediction models in our cohorts. Another reason could be that compared to the other studies—except the ECMOnet-Score, which only refers to the very specific H1N1 population and included significantly healthier patients—we identified as predictors only extrapulmonary variables which in addition to ARDS detrimentally influence outcome.

All 108 ARDS patients were treated with an identical ECMO system (Cardiohelp®; Maquet, Getinge Group) which was only approved in 2009, so that patients are well matched and good data and results could be achieved despite the limited sample size of our derivation group. Compared to the existing prediction scores derived from other studies, we included a similar number of patients (ECMOnet-Score, 60 patients; Roch-Score, 85 patients; PRESERVE-Score, 140 patients) and each patient received similar treatment regimens. This is a strength of the present study since avoiding technical heterogeneity makes results very comparable.

Furthermore, we had a heterogeneous patient population with a wide range of disease patterns. Thus, the novel PRESET-Score is likely applicable to a broad range of ARDS-evoked ECMO candidates and not limited to a specific underlying disease.

In contrast, the ECMOnet-Score reflects a young and more homogeneous population (82% H1N1 patients) and there were various contraindications, potentially creating a bias through exclusion of patients with a poor prognosis. Interestingly, subanalysis of our patients with H1N1 only demonstrated a better discrimination using the ECMOnet-Score, indicating that the ECMOnet-Score may be more appropriate in cohorts with H1N1 patients.

The potential practical usefulness of the new score should be emphasized. We created and validated in two independent cohorts an easy-to-use score covering a broad range of ARDS patients. In the derivation cohort, an ICU mortality of 93% was found in patients with a PRESET-Score ≥ 10 (risk class III; Fig. 1a). Furthermore, the internal validation cohort showed an ICU mortality of 91% in the same risk class, which raises the question of whether initiation of ECMO therapy in these patients is still reasonable.

On the other hand, patients with a low (≤5) PRESET-Score showed a 74% survival rate in the derivation cohort (risk class I; Fig. 1a) and 86% in the internal validation cohort, respectively, assuming that these patients are good candidates for ECMO therapy when conventional therapy is unsuccessful. Here, the PRESET-Score might indeed serve as a good tool in decision-making, especially when resources are limited.

### Limitations

This is a retrospective study performed at a single medical centre. Therefore, the results and the prognostic relevance of the PRESET-Score may not be directly applicable to other institutions harbouring patients with different ARDS aetiologies.

The aim of our study was the prediction of mortality before ECMO initiation and no extended follow-up addressing 6-month/long-term survival, quality of life, or permanent disability was conducted. This may be considered a limitation.

Furthermore, although the PRESET-Score is a useful prediction model, it should only supplement individual judgement based on history, condition, prognosis, and the assumed living will of any specific patient. Beyond scores, weighing treatment options requires experienced physicians but our score serves as an additional block to build and facilitate decision-making.

## Conclusions

We propose and validated prospectively a novel but simple prediction model to be used prior to ECMO initiation which also incorporates extrapulmonary variables, the PRESET-Score. This score better predicts mortality than previous scores, is easy to implement in clinical routine, and may guide decision-making when considering ECMO therapy.
